# Concurrent Brucellosis and Acute Lymphoblastic Leukemia: A Case Report

**DOI:** 10.1155/crh/1752123

**Published:** 2026-07-07

**Authors:** Parisa Bahramian, Marziyeh Ghalamkari, Mahdi Khatuni, Saeed Kalantari, Behnaz Varaminian

**Affiliations:** ^1^ Hematologist and Medical Oncologist, Tehran University of Medical Sciences, Tehran, Iran, tums.ac.ir; ^2^ Hematologist and Medical Oncologist, Iran University of Medical Sciences, Tehran, Iran, iums.ac.ir; ^3^ Pulmonologist, Shahid Beheshti University of Medical Sciences, Tehran, Iran, sbmu.ac.ir; ^4^ Antimicrobial Resistance Research Center, Iran University of Medical Sciences, Tehran, Iran, iums.ac.ir; ^5^ Hematologist and Medical Oncologist, Semnan University of Medical Sciences, Tehran, Iran, semums.ac.ir

**Keywords:** acute lymphoblastic leukemia, brucellosis, case report, Iran, Philadelphia chromosome

## Abstract

**Introduction:**

Acute lymphoblastic leukemia (ALL) is a rare hematologic malignancy that leads to significant qualitative and quantitative lymphocyte deficits. These immunological gaps predispose patients to various bacterial and opportunistic infections. Brucellosis, an intracellular infection endemic in many regions including Iran, presents with nonspecific symptoms such as prolonged fever, malaise, and organomegaly, which closely mimic the clinical manifestations of leukemia. Their co‐occurrence, even in brucellosis‐endemic areas, is actually rare. In addition, their similarity in clinical presentation can result in diagnostic delay or misdiagnosis.

**Case Presentation:**

A 47‐year‐old male shepherd from an endemic region in Iran presented with a 1‐month history of progressive weight loss, fever (38.4°C), and muscle cramps. Initial physical examination revealed pallor and tachycardia without lymphadenopathy. Laboratory investigations showed severe leukocytosis (103 × 10^3^/μL) and elevated lactate dehydrogenase (1190 U/L). Although initial serological tests for brucellosis (Wright and 2‐ME) were negative, bone marrow aspiration and flow cytometry confirmed B‐cell ALL. Cytogenetic analysis identified the Philadelphia chromosome (*t* (9; 22), p210 transcript). The patient began induction chemotherapy with the hyper‐CVAD regimen combined with dasatinib. Despite hematological improvement, persistent fever and diaphoresis prompted a re‐evaluation for infectious etiologies. Repeated brucellosis testing yielded strongly positive results (Wright 1/320 and 2‐ME 1/640), suggesting a potential prozone effect in initial tests or recent acquisition. Antibiotic therapy with rifampin and doxycycline was integrated into the oncological protocol. The patient showed rapid clinical improvement within 1 week and subsequently achieved complete remission, which was maintained throughout a 2‐year follow‐up.

**Conclusion:**

This case highlights the importance of considering brucellosis in the differential diagnosis of febrile patients with hematologic malignancies in endemic areas. Overlapping symptoms can lead to diagnostic delays. It is crucial to repeat serological tests or use serial dilutions to circumvent the prozone effect when clinical suspicion persists in immunocompromised patients.

**Learning Points**
• Coincidence of brucellosis and ALL is so rare.
• The similarity of clinical symptoms of *Brucella* and ALL can cause a diagnostic delay.
• Treatment of both of them should be started concomitantly.

## 1. Introduction

Acute lymphoblastic leukemia (ALL), a rare hematologic malignancy, results in qualitative lymphocyte deficits, leading to hypogammaglobulinemia and reduced cell‐mediated immunity that predispose patients to certain bacterial, viral, and fungal infections [1].

The clinical presentation of ALL is nonspecific, including symptoms such as prolonged fever, cytopenia, and organomegaly [2]. These presentations are difficult to distinguish from ordinary infectious diseases [3].

*Brucella* is an intracellular, Gram‐negative microorganism; the main clinical presentations of brucellosis are fever, malaise, and night sweats, which may resemble malignancies such as ALL [4].

There have been rare cases of brucellosis in ALL patients [2]. Here, we report a patient who had ALL and, during induction treatment, brucellosis was diagnosed.

## 2. Case Presentation

A 47‐year‐old Iranian man presented to the clinic with complaints of progressive weight loss, constitutional symptoms, and generalized muscle cramps during the past month.

He was a shepherd who worked in Qazvin Province, Iran. According to his job and place of residence, brucellosis diagnostic tests (Wright and 2‐ME) were performed, and the results were initially negative.

On physical examination, he was obviously pale, febrile (axillary temperature: 38.4°C), and tachycardic (pulse rate = 102/minute), with normal blood pressure and respiratory rate.

He had no lymphadenopathy or organomegaly.

The laboratory findings are summarized in Table 1.

**TABLE 1:** Patient laboratory findings at presentation.

	At presentation	Reference value
White blood cells (10^3^/μL)	103	4–11
Neutrophils (10^3^/μL) [%]	64.8 [63%]	
Band (10^3^/μL)	12.36 [12%]	
Myelocyte (10^3^/μL)	8.24 [8%]	
Metamyelocytes (10^3^/μL)	3.09 [3%]	
Lymphocytes (10^3^/μL)	13.39 [13%]	
Eosinophils (10^3^/μL)	1.03 [1%]	
Hemoglobin (g/dL)	11.5	13–17.5
MCV (fL)	79	80–100
Platelets (10^3^/μL)	126	140–450
CRP (mg/L)	18	< 10
ESR (mm/hour)	43	0–20
Sodium (mg/dL)	140	150–350
Potassium (mg/dL)	3.9	3.5–5.5
Creatinine	1.2	
Aspartate transaminase (AST) (U/L0	19	< 37
Alanine transaminase (ALT) (U/L)	27	< 40
Total bilirubin	0.2	< 2
Direct bilirubin	0.1	
Lactate dehydrogenase (LDH) (U/L)	1190	< 480
Blood culture	Negative	
Urine culture	Negative	
Wright	Negative	

**FIGURE 1:**
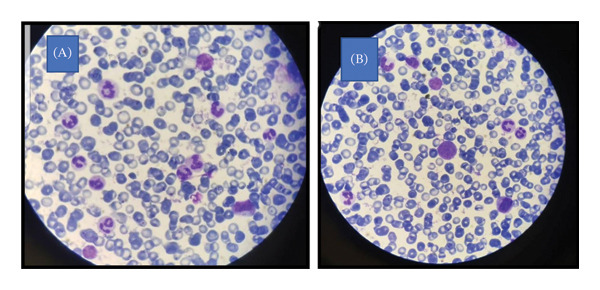
Peripheral blood smear (^∗^10) (A) and (B): leukocytosis with shift to left and significant lymphoblast cells.

A peripheral blood smear was performed (Figure 1) and revealed leukocytosis with a shift to the left and suspicious blast cells.

Bone marrow aspiration (BMA) and biopsy were performed.

Flow cytometry of BMA was compatible with B‐cell ALL.

Karyotype study in bone marrow culture: 47, XY, *t* (9; 22) (q34; q11) (20 metaphases).

Qualitative BCR‐ABL (p190‐210) testing: negative for BCR‐ABL (p190) transcript and positive for BCR‐ABL (p210) transcript.

Finally, based on all the above findings, the patient was diagnosed as ALL with Philadelphia chromosome–positive. He underwent induction chemotherapy with hyper‐CVAD regimen (cyclophosphamide, mesna, vincristine, doxorubicin, and dexamethasone) and received oral tyrosine kinase inhibitor (dasatinib).

After chemotherapy initiation, the patient’s white blood cell counts decreased, but he still suffered from fever and severe diaphoresis.

*Brucella* tests were repeated based on his occupational exposure. Unexpectedly, the results were positive:

- Wright test = 1/325
- 2 ME (2‐mercaptoethanol) = 1/640

Finally, confirming the diagnosis of brucellosis, the treatment (antibiotic therapy: rifampin 600 mg daily and doxycycline 100 mg BID) was added to the chemotherapy. The patient’s symptoms and signs resolved within 1 week, and he tolerated the therapy well. The antibiotics for brucellosis continued for 3 months without any adverse effects. The 2‐ME titer was negative after 6 months. No probiotics were administered to him during this time.

Following induction chemotherapy with the hyper‐CVAD regimen plus dasatinib, the patient achieved complete remission. Subsequently, he underwent a 2‐year maintenance therapy consisting of vincristine, dexamethasone, oral methotrexate, mercaptopurine, and dasatinib.

The patient remains in complete clinical and hematological remission as of the last follow‐up.

## 3. Discussion

Patients with hematologic malignancies are at an increased risk of infections, which are associated with significant morbidity and mortality. Patients with acute myeloid leukemia (AML) exhibit qualitative and quantitative deficits in granulocytes, predisposing them to bacterial and fungal infections. Similarly, ALL results in qualitative lymphocyte deficits, characterized by hypogammaglobulinemia and reduced cell‐mediated immunity, which predisposes patients to specific bacterial, viral, and fungal infections. Chemotherapeutic regimens often compound these deficits, resulting in prolonged periods of severe neutropenia and disrupted mucosal barriers, further elevating the risk of infections [4, 5].

Brucellosis, an intracellular bacterial disease, is acquired by humans through direct contact with infected animals, consumption of contaminated animal products, or inhalation of airborne agents. Most cases result from ingesting unpasteurized milk or cheese from infected goats or sheep; notably, human‐to‐human transmission is exceedingly rare [6, 7].

Patients with malignancies may present with atypical symptoms of brucellosis, a condition that remains rare even in endemic regions. Clinicians should be aware of its potential impact on cancer treatment; as if the patient’s immune level decreases as a result of cancer therapy, it may potentially trigger the reactivation or worsening of latent brucellosis complications. Consequently, brucellosis should be considered in the differential diagnosis of febrile neutropenia in cancer patients, particularly in endemic countries [8].

The relationship between brucellosis and ALL is exceptionally rare, even in endemic countries like Iran [1]. In our case, the reason for the initial negative *Brucella* diagnostic tests remains unclear. One possibility is the lower sensitivity of laboratory kits in smaller medical centers. Another possibility is that the *Brucella* infection was acquired in the interval between the first set of tests and the subsequent ones performed at our hospital. Alternatively, the prozone effect may be responsible; this occurs in the presence of excessively high antibody titers and requires the samples to be retested using higher serial dilutions [9].

Due to the overlapping clinical symptoms of ALL and brucellosis, the patient was initially admitted for severe leukocytosis and other findings suggestive of leukemia, and *Brucella* tests were not immediately repeated. However, as the clinical symptoms did not fully resolve during treatment, repeat testing yielded positive results. This delayed diagnosis may be attributed to the overlapping clinical manifestations or a technical error. It is crucial to repeat diagnostic tests for suspected infections in immunocompromised patients when clinical suspicion persists.

## Funding

No funding was received for this manuscript.

## Disclosure

All authors have read and approved the final version of the manuscript. The corresponding author “Marziyeh Ghalamkari” had full access to all of the data in this study and takes complete responsibility for the integrity of the data and the accuracy of the data analysis.

## Consent

We confirm that this study was conducted with ethical approval of the patient. Written informed consent was obtained from the patient for publication of this case report and any accompanying images.

## Conflicts of Interest

The authors declare no conflicts of interest.
